# Telerehabilitation Policy Report: Interprofessional Policy Principles and Priorities

**DOI:** 10.5195/ijt.2021.6433

**Published:** 2021-12-16

**Authors:** Evelyn Abrahante Terrell, Andrew Bopp, Kristen Neville, David Scala, Kyle Zebley

**Affiliations:** 1 Director of Telehealth and Special Projects, Nicklaus Children's Hospital, Miami, Florida, USA, & Chair of American Telemedicine Association's Telerehabilitation Special Interest Group; 2 Senior Legislative Representative, American Occupational Therapy Association, North Bethesda, Maryland, USA; 3 Manager, State Affairs, American Occupational Therapy Association, North Bethesda, Maryland, USA; 4 Senior Specialist, Congressional Affairs, American Physical Therapy Association, Alexandria, Virginia, USA; 5 Vice President, Public Policy, American Telemedicine Association, Arlington, Virginia, USA

**Keywords:** Policy, Rehabilitation services, Telehealth, Telepractice, Telerehabilitation

## Abstract

The American Occupational Therapy Association, the American Physical Therapy Association, the American Speech-Language-Hearing Association and the American Telemedicine Association are collaborating to advance telehealth and ensure sustainability of virtual care services beyond the COVID-19 pandemic. These professional associations represent the interests of more than 888,000 rehabilitation services professionals. This paper summarizes the current state of telehealth policy principles and priorities for rehabilitation services. The report outlines key considerations when advocating with policymakers to avoid the “Telehealth Cliff” for audiology and therapy services and to facilitate the continued advancement of telehealth innovation and transformation by rehabilitation services professionals.

The COVID-19 pandemic has transformed rehabilitation service delivery and consequently impacted how health care providers, professional associations, and their members have implemented telehealth and telepractice. Rehabilitation services professionals include occupational therapists, occupational therapy assistants, physical therapists, physical therapy assistants, speech-language pathologists, speech-language pathology assistants, audiologists, and other members of the care team who serve individuals across the lifespan in all health care settings, facilities, and specialty areas. Telehealth reduces geographic and access barriers, and addresses critical workforce shortage of professionals. Virtual care has been shown to improve patient satisfaction and experiences and addresses health equity challenges; these are paramount to ensuring optimal outcomes and access to quality services for all people (Bican et al., 2021; Krasovsky et al., 2021; Little et al., 2021).

Science-driven, evidence-based telehealth interventions by rehabilitation services professionals have enabled people of all ages to develop, regain, and build functional communication, mobility, and independence in everyday activities, to live life to its fullest. Telehealth has improved access and been an invaluable option for individuals with chronic conditions, and those residing in rural/underserved areas, lacking transportation, or facing health care disparities and other socioeconomic barriers.

The American Occupational Therapy Association (AOTA), the American Physical Therapy Association (APTA), the American Speech-Language-Hearing Association (ASHA) and the American Telemedicine Association (ATA) collaborated to advance telehealth access during Telehealth Awareness Week (TAW), September 19–25, 2021. During TAW a significant milestone was achieved: a bipartisan, bicameral resolution was introduced in both the U.S. House and Senate recognizing September 19-25 as TAW. The Senate unanimously passed the resolution, sending an important signal that they essentially agreed telehealth is here to stay.

The aforementioned professional organizations are working with policymakers to support legislation that will promote access to care, mitigate risks associated with COVID-19, and ensure sustainability of telehealth services beyond the pandemic. These national and international professional associations are the leading organizations representing the interests of more than 888,000 rehabilitation services professionals in the US combined. AOTA, APTA, ASHA, and the ATA have shared priorities that are multi-pronged and include legislative, regulatory, and state-based advocacy efforts. The organizations have collaborated to develop, support, or endorse legislation to assist members in sustaining telehealth services and hybrid models of care that include in-person and virtual care. Their efforts focus on specific priorities:

- Advocating with state and federal policymakers to advance permanent telehealth reform that does not restrict provider type, supports enhanced provider autonomy, and ensures access to non-physician providers.
- Supporting the bipartisan Expanded Telehealth Access Act (H.R.2168), which would expand the type of practitioners eligible to offer telehealth by adding audiologists, occupational therapists, physical therapists, and speech-language pathologists. This legislation would allow practitioners to become permanent authorized providers of telehealth under the Medicare program. We are urging Congress to remove restrictions on where the patient must be located to receive health care access, and support provisions that would enable the patient's home to serve as the originating site for telehealth.
- Insisting that federal law should not dictate service restrictions like patient location, specific technology, modality of care, provider type, and is in support of some audio-only services.
- Supporting licensure compacts and portability to enable practice across state borders, as well as advocating in support of expanding telehealth authorization by state licensing boards.
- Supporting coverage by Medicaid and commercial insurers of therapy services provided via telehealth.
- Supporting equitable payment for telehealth services (AOTA, 2021; APTA, 2021; ASHA, 2021; ATA, 2021a).

Congressional action is essential to avoid the “Telehealth Cliff” for audiology and therapy services. If Congress does not act before the end of the COVID-19 public health emergency (PHE), access to care could be abruptly cut-off. Medicare beneficiaries would lose access to critical virtual care options, which have become a lifeline to many, especially for underserved and rural populations (ATA, 2021b). Congressional action is also imperative to facilitate the continued advancement of telehealth innovation and transformation by rehabilitation services professionals.
